# Delegating Clozapine Monitoring to Advanced Nurse Practitioners: An Exploratory, Randomized Study to Assess the Effect on Prescription and Its Safety

**DOI:** 10.1007/s10488-020-01031-4

**Published:** 2020-03-18

**Authors:** Y. C. van der Zalm, P. F. Schulte, J. P. A. M. Bogers, F. Termorshuizen, M. Marcelis, M. A. G. B. van Piere, I. E. Sommer, J. P. Selten, CLOZ-NP Study Group

**Affiliations:** 1 Rivierduinen Institute for Mental Health, Leiden, The Netherlands; 2 Department of Psychiatry & Neuropsychology, School for Mental Health and Neuroscience, Maastricht University Medical Center, Maastricht, The Netherlands; 3 Dutch Clozapine Collaboration Group, Castricum, The Netherlands; 4 Mental Health Service Noord-Holland Noord, Alkmaar, The Netherlands; 5 Institute for Mental Health Care Eindhoven (GGzE), Eindhoven, The Netherlands; 6 Department of Neuroscience and Department of Psychiatry, University Medical Center Groningen, Groningen, The Netherlands

**Keywords:** Clozapine, Treatment-resistant schizophrenia, Underutilization, Outpatients, Randomized trial, Nurse practitioner

## Abstract

To test whether: (1) psychiatrists will prescribe clozapine more often if they can delegate the monitoring tasks to an advanced nurse practitioner (ANP), (2) clozapine monitoring by an ANP is at least as safe as monitoring by a psychiatrist. Patients from 23 Dutch outpatient teams were assessed for an indication for clozapine. ANPs affiliated to these teams were randomized to Condition A: clozapine monitoring by an ANP, or Condition B: monitoring by the psychiatrist. The safety of monitoring was evaluated by determining whether the weekly neutrophil measurements were performed. Staff and patients were blinded regarding the first hypothesis. Of the 173 patients with an indication for clozapine at baseline, only seven in Condition A and four in Condition B were prescribed clozapine (Odds Ratio = 2.24, 95% CI 0.61–8.21; p = 0.225). These low figures affected the power of this study. When we considered all patients who started with clozapine over the 15-month period (N = 49), the Odds Ratio was 1.90 (95% CI 0.93–3.87; p = 0.078). With regard to the safety of the monitoring of the latter group of patients, 71.2% of the required neutrophil measurements were performed in condition A and 67.3% in condition B (OR = 0.98; CI = 0.16–3.04; p = 0.98). Identifying patients with an indication for clozapine does not automatically lead to improved prescription rates, even when an ANP is available for the monitoring. Clozapine-monitoring performed by an ANP seemed as safe as that by a psychiatrist.

**Electronic supplementary material:**

The online version of this article (10.1007/s10488-020-01031-4) contains supplementary material, which is available to authorized users.

## Introduction

Despite evidence for the superiority of clozapine as therapy for treatment-resistant Non-Affective Psychotic Disorder (NAPD) (Kane et al. 1988; Siskind et al. 2016; Souza et al. 2013), its prescription rate remains low (Bachmann et al. 2017; Stroup et al. 2014) and clozapine initiation is often delayed (Grover et al. 2015; Howes et al. 2012; Ucok et al. 2015). This delay unnecessarily prolongs patients’ suffering and impedes their recovery. Moreover, there is some evidence that a delay may even diminish efficacy of clozapine (Ucok et al. 2015). Important reasons for this delay and under-prescription may be concerns about the safety of clozapine and the need for regular laboratory investigations to prevent potentially dangerous side-effects (Gee et al. 2014; Nielsen et al. 2010; Tungaraza and Farooq 2015). More specifically, the mandatory weekly neutrophil measurements in the first months, to detect agranulocytosis, and the regular monitoring of other side-effects are time consuming and a burden to both patients and doctors. In a survey, UK professionals considered the deployment of dedicated staff to arrange and monitor this initiation phase as the factor most likely to increase the prescribing of clozapine (Gee et al. 2014). The establishment of specialised teams for the management of patients with treatment-resistant schizophrenia including clozapine treatment in London, increased the number of patients who started to use this drug (Beck et al. 2014). However, the authors acknowledged that there are disadvantages to deploying additional teams: an extra service can cause confusion among clinicians and patients about the clinicians’ role and responsibilities, because patients have multiple appointments with different teams of health professionals. With a view to stimulating clozapine use, the aims of this study were to test the following hypotheses: (1) psychiatrists prescribe clozapine more often if they can delegate the monitoring tasks to an advanced nurse practitioner (ANP); (2) monitoring by an ANP is at least as safe as monitoring by a psychiatrist; and (3) delegation of monitoring tasks to an ANP is associated with less frequent premature termination of clozapine in the initial phase (first 18 weeks).

## Methods

### Setting/Design

This exploratory study, set up as a cluster-randomized trial (study registration NTR5135), involved Dutch outpatient teams for patients with Non-Affective Psychotic Disorder (NAPD), called Flexible Assertive Community Treatment (FACT) teams. These teams treat patients with severe mental illness and are flexible in that treatment can be intensified in order to prevent the hospitalization of patients during a crisis (van Veldhuizen 2007). FACT teams are responsible for a specific area and their caseload consists of approximately 200–250 outpatients, most of whom have NAPD. In some areas, there are also Early Intervention Teams, which treat patients up to 5 years after the first onset of psychosis. These teams differ from FACT teams in that their caseloads are smaller and the patients younger. While teams typically include a psychiatrist, not all teams have an ANP. After at least 2 years of experience in psychiatry, Dutch nurses can follow a 2- or 3-year training programme to become an ANP in mental health care. The profession of ANP in mental health care in the Netherlands resembles that of a mental health ANP in for example the UK, France and Australia and that of a Psychiatric Mental Health Nurse Practitioner in the USA, although in some countries they are authorized to prescribe drugs and in other countries not. In this study, they did not prescribe clozapine. Given the objective of this study, only teams with an ANP were included.

### Procedures

Before randomization, in order to prevent bias, the authors trained the ANPs and psychiatrists of all participating teams for 3 h about indications for clozapine and monitoring guidelines. Subsequently, the ANP and psychiatrist of each team assessed whether patients had an unmet indication for clozapine, using a standardised procedure (van der Zalm et al. 2018). The decision tree used during this procedure is shown in Online Appendix. The principle investigator (PI) was present at this discussion and available for advice. Thereafter, the ANPs with their corresponding teams were randomized to one of two conditions: (A) intervention condition: the ANP performed the somatic screening of patients before clozapine was started, the psycho-education of the patients and their relatives, and the monitoring of laboratory investigations and side-effects; where necessary, they asked supervision from the psychiatrist; or (B) treatment as usual: the psychiatrist performed these tasks. In both conditions, the psychiatrist was responsible for the decision to start clozapine and for prescribing it. In order to avoid the assignment of an ANP to both conditions, we decided to randomize the ANPs instead of the teams. Psychiatrists, ANPs, and patients were kept blind to the first hypothesis about the number of patients that would start to use clozapine in each condition. They were only aware of the other two research questions. The randomization was stratified by hospital, geographical area, and FACT vs. Early Intervention Team.

In September 2015, the ANPs randomized to condition A were trained by psychiatrists of the Dutch Clozapine Expert Group and a mental health ANP in two sessions of 3 h each. The topics covered were: (1) laboratory investigations—their frequency, the interpretation of the results, and the necessary or recommended actions to be taken; (2) dangerous side-effects of clozapine, such as agranulocytosis, myocarditis, and ileus, and how to prevent or detect them; (3) other side-effects such as sedation, orthostasis, constipation, hypersalivation, and metabolic syndrome and how to prevent or treat them; (4) possible interactions between clozapine and other drugs or tobacco use. The participants then had to pass a test of their knowledge.

All patients who started clozapine between 1 October 2015 and 1 January 2017 were included in this trial. The follow-up of each patient started at the moment of clozapine initiation and lasted 18 weeks, a period in which weekly neutrophil measurements are mandatory in the Netherlands. Patients who started clozapine when in hospital were also included, provided that they were discharged within 18 weeks. We excluded patients who started clozapine during hospital admission and who stayed there during the first 18 weeks. We assumed that for these patients, the decision to start was most often made by the responsible psychiatrist in the hospital. With reference to our second aim, about the safety of the monitoring, inpatient weeks of monitoring were excluded, because the focus of this study was on outpatient clozapine monitoring. The psychiatrist or the ANP informed the PI when clozapine was started. After 18 weeks, the PI visited the ANP or psychiatrist in his or her office. During this visit, the ANP or psychiatrist checked the medical file and provided the following information to the PI: blood assessments (dates and laboratory values) and duration of clozapine use (maximum of 18 weeks). The PI noted this information on structured forms. She asked explicitly for hazardous side-effects and, if clozapine use had been terminated, she documented the reasons for discontinuation. Within this context, it is unlikely that the ANP or psychiatrist invented or concealed outcomes.

### Measures

In order to assess the safety of clozapine monitoring, the PI determined whether the mandatory weekly neutrophil measurements had been performed and registered. If there was an interval of 9 or more days between laboratory investigations, she considered the measurement as missed. We reasoned that the number of missed measurements was an indication of the risk to which the patient was exposed. In addition, we checked the file for reports of dangerous side-effects (e.g. ileus, myocarditis, agranulocytosis, venous thromboembolism) and investigated how soon the ANP or psychiatrist alerted the relevant medical specialist.

### Statistical Analysis

Descriptive statistics were used to summarise demographic and clinical characteristics. We used multilevel logistic regression analysis to test for a difference in the proportion of patients who started to use clozapine. As a small number of patients without an NAPD diagnosis also started clozapine, we conducted one analysis with all patients treated by the teams at baseline, regardless of diagnosis, and another analysis restricted to those with an unmet indication for this drug at baseline. In these analyses, patient was the first level and team (the psychiatrist who could prescribe the drug) the second level.

We used a slightly different analysis to test for differences in the number of neutrophil measurements performed. In this analysis, the measurements were the first level, the individual patient the second level, and cluster (ANP or psychiatrist) the third level.

The difference in retention on clozapine was analysed using multilevel analysis, with patient as the first level and cluster (ANP or psychiatrist) as the second level. Duration of use was the dependent variable in this analysis. In an additional analysis, we compared the proportion of patients who stopped taking clozapine during the follow-up (χ^2^ test).

All multilevel analyses were random intercept models, adjusted for age, gender, and DSM-IV diagnosis (NAPD vs other diagnoses) as patient-level variables. The second analysis (of neutrophil measurements performed) was a model with random intercept and random slopes on patient level. This model was also adjusted for time (weeks) after clozapine initiation, because neutrophil measurements were more likely to be performed in the first weeks after treatment was started. Descriptive statistics were performed with SPSS, version 22.0. The multilevel analyses were performed with STATA, version 13.0, using procedure GLAMM. A p-value of < 0.05 was considered statistically significant for all tests.

We calculated the required sample size for a cluster-randomized trial with a power of 0.80 (one-sided testing, α = 0.05). We assumed that there would be at least 15 patients in each cluster with an unmet indication for clozapine (total n = 240), of whom on average 50% would actually start with this drug (N = 120). We also assumed that in our intervention condition twice as many patients would start with clozapine (OR = 2) and that the coefficient of intracluster correlation was 0.6. The results showed that we needed eight clusters in each condition (Hayes and Bennet 1999).

## Results

### Teams and Patients

**Fig. 1:**
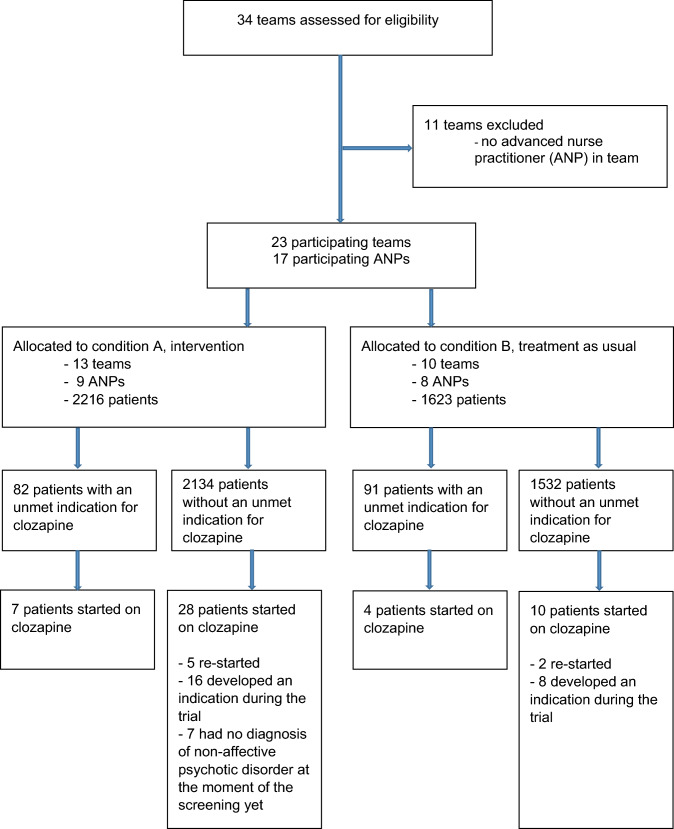
Flow diagram of teams and participants

Four psychiatric institutes in different Dutch regions agreed to participate in this trial. Of the five Early Intervention Teams and 29 FACT teams of these institutes, three Early Intervention Teams and 20 FACT teams were eligible, see Fig. 1. Seventeen ANPs worked for these 23 teams. Some ANPs worked for two teams, but there were no teams with more than one ANP. The ANPs were randomized into one of the two conditions: 9 ANPs, working for 13 teams, were assigned to condition A and 8 ANPs, working for ten teams, to condition B. At the start of the 15-month inclusion period, 3839 patients were being treated by these teams. There were no significant differences in mean age or gender between the patients of the two conditions, but there were minor differences in proportions of diagnoses. The baseline characteristics are shown in Table 1. Screening patients for an unmet indication for clozapine at baseline identified 82 patients in condition A (3.7% of all patients) and 91 patients in condition B (5.6% of all patients), see Fig. 1.

**Table 1:** Baseline characteristics of 3839 patients from 23 teams who participated in a cluster-randomized trial to compare clozapine monitoring by advanced nurse practitioners and psychiatrists

	Condition A*, intervention N = 2216	Condition B**, treatment as usual N = 1623	p***
Age, years: mean (S.D)	46.6 (12.4)	45.9 (12.6)	0.095
Male, n (%)	1353 (61.2)	1033 (63.7)	0.116
DSM-IV diagnosis, n (%)			.003
Schizophrenia	885 (39.9)	734 (45.3)	
Schizoaffective disorder	215 (9.7)	154 (9.5)	
Schizophreniform disorder	13 (0.6)	17 (1.0)	
Psychotic disorder not otherwise specified	367 (16.6)	258 (15.9)	
Other diagnosis/unknown	737 (33.2)	460 (28.3)	

^*^Condition A: delegation of clozapine-monitoring tasks to a trained advanced nurse practitioner

^**^Condition B: treatment as usual, clozapine monitoring by a psychiatrist

^***^χ^2^-test: age, gender, diagnosis (schizophrenia, schizoaffective disorder, schizophreniform disorder, psychotic disorder not otherwise specified, or other diagnosis)

### Prescription of Clozapine

Of the 173 patients with an unmet indication for clozapine, only 7 patients in condition A and 4 in condition B were started on clozapine (i.e. 6.4% of all patients with an unmet indication). The baseline characteristics of these patients are presented in Table 2. The odds ratio for starting clozapine in condition A compared to condition B, adjusted for age and gender was 2.24, CI 0.61–8.21; p = 0.225.

**Table 2:** Sociodemographic and clinical characteristics of patients who started clozapine in a cluster-randomized trial comparing clozapine monitoring by advanced nurse practitioners and psychiatrists

Characteristic	Starters with indication at baseline N = 11		Starters with indication at baseline or thereafter N = 49	
	Condition A*, intervention (n = 7)	Condition B**, treatment as usual (n = 4)	Condition A*, intervention (n = 35)	Condition B**, treatment as usual (n = 14)
Age, years: mean (S.D)	48.1 (3.1)	55.5 (6.4)	45.7 (12.3)	45.6 (14.0)
Male, n (%)	4 (57.1)	2 (50.0)	22 (62.9)	11 (78.6)
DSM-IV diagnosis, n (%)				
Schizophrenia	4 (57.1)	1 (25.0)	20 (57.1)	9 (64.3)
Schizoaffective disorder	2 (28.6)	2 (50)	3 (8.6)	2 (14.3)
Schizophreniform disorder			-	
Psychotic disorder not otherwise specified	1 (14.3)	1 (25.0)	7 (20.0)	3 (21.4)
Other diagnosis			5 (14.3)	

^*^Condition A: delegation of clozapine-monitoring tasks to a trained advanced nurse practitioner

^**^Condition B: treatment as usual, clozapine monitoring by a psychiatrist

^***^χ^2^-test: age, gender, diagnosis (schizophrenia, schizoaffective disorder, schizophreniform disorder, psychotic disorder not otherwise specified, or other diagnosis)

The reasons for not prescribing clozapine to patients were not systematically studied and this data was not recorded in the files. However, at baseline, psychiatrists and ANPs mentioned reasons for not prescribing clozapine to patients with an indication. A frequently mentioned reason was that they expected the patient not to collaborate with lab exams. Another frequently mentioned reason was non-compliance with oral medication in the past and therefore the need to stay on long-acting injectables. That the patient was doing much better than several years before and starting clozapine was not worth the risk, was also mentioned several times. Additional analysis on prescription of clozapine.

Apart from the patients with an unmet indication for clozapine at baseline, there were other patients in the teams who started with clozapine. Those patients either re-started the drug, developed an indication during the trial (due to an increase of positive symptoms or to a lack of effect of other antipsychotics), or did not have an NAPD diagnosis at baseline (see Fig. 1). In total, 49 started on clozapine during the study period: 35 in condition A and 14 in condition B. Table 2 shows the characteristics of these patients. Taking all 3839 patients into account, the odds ratio for starting clozapine in condition A compared to condition B, adjusted for age, gender and NAPD-diagnosis (yes/no) was 1.90 (95% CI 0.93–3.87; p = 0.078).

There were large differences between the teams in prescribing clozapine, see Supplementary Table S1. Psychiatrists who had spoken negatively about clozapine hardly prescribed it, regardless of the condition they were in. Conversely, psychiatrists with a strong positive attitude toward clozapine regularly prescribed it, also regardless of the condition they were in. On the other hand, some psychiatrists in condition A collaborating with three ANPs informed us that they were very glad that they were allocated to the intervention condition, because now they could start with ambulatory clozapine initiation. They stated that they had not prescribed clozapine if they had been allocated to condition B. Supplementary Table S1 shows the differences per team in patients on clozapine, with an indication for clozapine and who started with this drug. This table also shows that there were more patients newly admitted to the ambulatory team during the study period who started to use clozapine in condition A (N = 13) compared to condition B (N = 4). There was an in- and out-flow of patients during the inclusion period and it was not possible to keep track of all these changes. Nonetheless, differences between the conditions were in line with the first hypothesis.

### Safety of Clozapine Monitoring

For our second question on safety of clozapine monitoring, we included all patients who started clozapine in the participating teams (n = 49). In condition A, 8 patients started clozapine as an inpatient and another patient was admitted twice during the first 18 weeks. The mean duration of admission, for these 9 patients, was 6.9 weeks (SD 3.8). In condition B, 7 patients started clozapine as inpatients. Their mean duration of admission was 8.7 weeks (SD 4.9). After the exclusion of the weeks of inpatient treatment (mean 1.8 weeks in condition A and 4.4 weeks in condition B) and the time between premature stopping of clozapine and the end of follow-up, neutrophil measurement for the 49 included patients was mandatory for 682 weeks (517 in Condition A and 165 in condition B). Overall, 368 neutrophil measurements in condition A were performed as required (71.2%) and 111 in condition B (67.3%) (OR, adjusted for age, gender, and weeks after start 0.98; 95% CI, 0.16–6.04; p = 0.982). The proportion of neutrophil measurements carried out by one ANP or psychiatrist varied considerably. In condition A, this proportion ranged from 30.6 to 87.2% and in condition B from 0 to 97%. Supplementary Table 2 shows these proportions per cluster. No dangerous side-effects occurred in either condition. The reasons for missed neutrophil counts varied. In most cases the patients received a laboratory form, but did not go to the laboratory. In one particular area neutrophil measurements were missed because of a failing laboratory. For example, the wrong tests were performed or the laboratory assistant went to the wrong address. Holidays of patients were another reason for missed lab exams. A psychiatrist failed to notice that one patient missed all laboratory tests. Missing laboratory exams was only in one patient the reason to stop clozapine. This psychiatrist made the decision when the patient had a fever and persisted in refusing neutrophil measurements.

### Duration of Clozapine Use

For the analysis on duration of use, we also included all patients who started clozapine in the participating teams (n = 49). There were no significant differences in the retention on clozapine—the mean duration of use (including inpatient weeks of use)was 16.53 (SD 4.5) weeks in condition A and 15.96 (SD 3.4) weeks in condition B (b = 0.31; 95% CI −2.26–2.88; p = 0.815). In condition A, 11.4% of the clozapine starters stopped taking the drug prematurely (< 18 weeks) compared to 28.6% in condition B (χ^2^ = 2.15; df = 1; p = 0.142). One patient in condition B stopped to use clozapine after 7 weeks because it was not effective and one patient in condition A had to stop clozapine because of a fever in combination with the refusal to go to the laboratory. In all other patients (n = 6), the reason for discontinuation were the side-effects of clozapine. This was a shared decision for all patients except one.

## Discussion

### Main Findings

We tested the hypotheses that psychiatrists would prescribe clozapine more often if they could delegate the monitoring tasks to an ANP, that monitoring by an ANP is at least as safe as monitoring by a psychiatrist, and that delegation of monitoring tasks to an ANP is associated with a longer retention on clozapine. Our findings were consistent with the first hypothesis, but failed to reach the conventional level of statistical significance, most likely due to a lack of statistical power. The OR was close to the OR assumed in our power calculation, but the number of patients with an unfulfilled indication for clozapine was smaller than we expected. In addition, the number of patients who started with this drug in either condition were much smaller than expected. We conclude that even when an ANP is present for support, Dutch psychiatrists still fail to start clozapine for the vast majority of patients identified as having potential benefit from clozapine. We can only speculate about the causes of this hesitation. Possible reasons are the side-effects of clozapine, some of which are dangerous and require a prompt and adequate reaction, or an absence of trust in the potential benefits from this drug.

Clozapine monitoring by an ANP seems as safe, in terms of performed and recorded neutrophil measurements, as that done by a psychiatrist. Patients monitored by an ANP tended to stay on treatment for longer than patients monitored by a psychiatrist, but the difference was small and statistically not significant.

### Comparison with Other Studies

This study was the first randomized controlled trial to examine the effect of an intervention to stimulate the use of clozapine. The findings of our study are in line with those of the study of Goren et al. (2016). In their study, Goren et al. interviewed psychiatrists over the phone to identify facilitators of and barriers to clozapine use. They concluded that the involvement of ANPs and clinical pharmacists in clozapine teams was associated with high clozapine prescription rates. This multidisciplinary approach is comparable to the ANP condition in our study, where all ANPs collaborated with a psychiatrist. As for the mandatory weekly neutrophil measurements, it is difficult to compare the results between different settings. Of note, in the Netherlands, there is no manufacturer-organised mandatory service or database for haematological monitoring. It is the responsibility of the physician to organise these weekly laboratory investigations. To our knowledge, only one other study reported the frequency of neutrophil measurements after the initiation of clozapine, with measurements being performed during the first 18 weeks at a mean interval of 25 days (Ingimarsson et al. 2016). This is less often than in our study.

In order to compare sole nurse-led clozapine services to physician-led teams, Gage et al. (2015) interviewed patients and concluded that clinics run by a nurse could effectively provide clozapine-monitoring services. However, the lack of direct access to a physician led to an increased use of community psychiatric services and to more hospital psychiatrist appointments. This argues for a multidisciplinary approach within one team, as occurred in the ANP condition in our study.

### Strengths and Limitations

A strength of this study is that all patients, psychiatrists, and ANPs were (and remained) blind to the first hypothesis. Another strength is that this real-world study involved patients and healthcare professionals from a non-academic setting, which is representative for many European services. Additionally, multidisciplinary outpatient teams like the FACT-teams in the Netherlands are comparable to services in other European countries (Rosenheck et al. 2016; Valdes-Stauber et al. 2014). However, some limitations need to be addressed. First, since the proportion of patients starting clozapine was smaller than expected, especially among those with an indication at baseline, the power of the trial to address the research questions was insufficient. Although we did not approach our second question as a non-inferiority analysis, the results do not indicate that the monitoring in our intervention condition was less safe. Second, the appraisal of the safety of the monitoring was limited to the number of neutrophil measurements performed and to the reporting of dangerous side-effects in the patient files. Information on whether the results of the laboratory investigations were checked in time is usually not recorded. It was not possible to investigate whether other aspects, such as constipation and blood pressure, were monitored as required by guidelines. Third, the data-collection was not performed by blinded research assistants. We believe that asking permission for an independent researcher to check the file, would have lowered the number of participants, because many patients are hesitant to start on clozapine and some of them are paranoid. In order to prevent bias, the PI was present at the moment the ANP or the psychiatrist checked the files for the data-collection. Fourth, in condition A, there was a collaboration between ANP and psychiatrists, which may have been an advantage. However, since ANPs cannot be responsible for the total of clozapine care, a small involvement of a psychiatrist, as in our condition A, corresponds to reality. Fifth, the training of psychiatrists and ANPs preceding the trial and the assessment of patients for an unmet indication for clozapine, could have increased the number of patients to start with clozapine. However, the number of patients that started was much lower than expected and this effect should be the same for both conditions. In addition, the knowledge of being in a study on safety of clozapine monitoring may have increased the number of lab exams. Since both psychiatrists and ANPs indicated that they did not want to be inferior to those in the other condition, we expected this effect to be similar in the conditions. Sixth, we were unable to adjust for the availability of a point-of-care (POC) device to test neutrophils, because only one team was in possession of such a device at the start of the follow-up (a team in condition B). Bogers et al. (2015) found that patients preferred POC testing and that this method moderately influenced their motivation for clozapine therapy. The availability of POC testing could, therefore, have led to more patients starting with clozapine and to a longer retention. Finally, the results of this study are only generalizable to countries where prescribers are responsible for clozapine monitoring, so without an independent clozapine monitoring agency.

### Implications

The results of this trial show that identifying patients with an indication for clozapine does not automatically lead to improved prescription rates. The results also suggest that some prescribers do not prescribe clozapine, irrespective of the condition they were in. In future research on interventions to stimulate use of clozapine, the attitude of the prescriber may be a better target for interventions. However, given the odds ratio and the p-value found in this small sample, we are confident that the use of clozapine can be stimulated by delegating the labour-intensive monitoring tasks to an ANP without compromising safety. This strategy can lead to earlier recovery from chronic psychosis and better patient outcome.

## Electronic supplementary material

Below is the link to the electronic supplementary material.

Supplementary file1—Supplementary Table S1 (DOCX 14 kb)

Supplementary file2—Supplementary Table S2 (DOCX 13 kb)

Supplementary file3—Online Appendix (DOCX 43 kb)
