# Supplementary material for: Delegating Clozapine Monitoring to Advanced Nurse Practitioners: An Exploratory, Randomized Study to Assess the Effect on Prescription and Its Safety
Source: Adm Policy Ment Health. 2020 Mar 18;47(4):632–40. doi: 10.1007/s10488-020-01031-4 (PMC7253396; doi:10.1007/s10488-020-01031-4)
Supplement: Supplementary file 1 — Supplementary file1—Supplementary Table S1 (DOCX 14 kb) [file 10488_2020_1031_MOESM1_ESM.docx]

Supplementary Table S1. The numbers of patients with an indication for clozapine and of those who started with this drug.

| Condition A*, intervention | Patients, any diagnosis  N=2216 | Patients with NAPD**  N=1451 | On clozapine  N=319 (%) | With indication for this drug  N=82 (%) | Patients started on clozapine  N=35 (%) | Started with indication at baseline.  N=7 | Newly admitted patients who started (not included in study)  N=13 |
| --- | --- | --- | --- | --- | --- | --- | --- |
| ANP 1 | 174 | 122 | 26 (21) | 9 (7) | 5 (4) | 1 | - |
| ANP 2 | 191 | 117 | 24 (21) | 2 (2) | 1 (1) | - | - |
| ANP 3 | 334 | 194 | 63 (32) | 4 (2) | 2 (1) | - | 1 |
| ANP 4 | 391 | 211 | 32 (15) | 15 (7) | 11 (5) | 1 | 3 |
| ANP 5 | 215 | 125 | 22 (18) | 4 (3) | 4 (3) | - | 1 |
| ANP 6 | 332 | 281 | 74 (26) | 16 (6) | 9 (3) | 3 | 1 |
| ANP 7 | 346 | 267 | 63 (24) | 24 (9) | 3 (1) | 2 | 6 |
| ANP 8 | 145 | 91 | 10 (11) | 7 (8) | - | - | 1 |
| ANP 9 *** | 88 | 71 | 6 (8) | 1 (1) | - | - | - |

| Condition B**.  treatment as usual | N=1623 | N=1163 | N=243 (%) | N=91 (%) | N=14 (%) | N=4 | N=4 |
| --- | --- | --- | --- | --- | --- | --- | --- |
| Psychiatrist 1 | 183 | 134 | 37 (28) | 16 (12) | 4 (3) | 2 | - |
| Psychiatrist 2 | 216 | 129 | 24 (19) | 20 (16) | 2 (2) | 1 | 1 |
| Psychiatrist 3 | 170 | 91 | 13 (14) | 6 (7) | 1 (1) | 1 | - |
| Psychiatrist 4 *** | 102 | 92 | 13 (14) | - | 2 (2) | - | 1 |
| Psychiatrist 5 | 488 | 375 | 95 (25) | 21 (6) | 1 (0.2) | - | - |
| Psychiatrist 6 *** | 167 | 140 | 15 (11) | 14 (10) | 2 (1) | - | - |
| Psychiatrist 7 | 146 | 98 | 18 (18) | 10 (10) | 2 (2) | - | 1 |
| Psychiatrist 8 | 151 | 104 | 28 (27) | 4 (4) | - | - | 1 |

* Condition A: delegation of clozapine-monitoring tasks to a trained advanced nurse practitioner. Condition B: treatment as usual, clozapine monitoring by a psychiatrist.

** Non Affective Psychotic Disorder

*** Early intervention team
