# Supplementary material for: Delegating Clozapine Monitoring to Advanced Nurse Practitioners: An Exploratory, Randomized Study to Assess the Effect on Prescription and Its Safety
Source: Adm Policy Ment Health. 2020 Mar 18;47(4):632–40. doi: 10.1007/s10488-020-01031-4 (PMC7253396; doi:10.1007/s10488-020-01031-4)
Supplement: Supplementary file 2 — Supplementary file2—Supplementary Table S2 (DOCX 13 kb) [file 10488_2020_1031_MOESM2_ESM.docx]

Supplementary Table 2. The numbers of patients who started on clozapine and the numbers of (mandatory and performed) neutrophil measurements, per condition and by each advanced nurse practitioner (Condition A) and psychiatrist (Condition B).

| Condition A*, intervention | Patients started on clozapine  N=35 | Mandatory neutrophil measurements  N=517 | Neutrophil measurements, performed on time (%)  N=368 (71.2) |
| --- | --- | --- | --- |
| ANP 1 | 5 | 77 | 66 (85.7) |
| ANP 2 | 1 | 14 | 7 (50) |
| ANP 3 | 2 | 36 | 11 (30.6) |
| ANP 4 | 11 | 157 | 114 (72.6) |
| ANP 5 | 4 | 42 | 15 (35.7) |
| ANP 6 | 9 | 144 | 114 (79.2) |
| ANP 7 | 3 | 47 | 41 (87.2) |
| ANP 8 | - | - | - |
| ANP 9 | - | - | - |

| Condition B**.  treatment as usual | Patients started on clozapine  N=14 | Mandatory neutrophil measurements  N=165 | Neutrophil measurements, performed on time (%)  N=111 (67.3) |
| --- | --- | --- | --- |
| Psychiatrist 1 | 4 | 38 | 26 (68.4) |
| Psychiatrist 2 | 2 | 38 | 34 (89.5) |
| Psychiatrist 3 | 1 | 9 | 1 (11.1) |
| Psychiatrist 4 | 2 | 27 | 20 (74.1) |
| Psychiatrist 5 | 1 | 5 | 0*** (0) |
| Psychiatrist 6 | 2 | 19 | 2 (10.5) |
| Psychiatrist 7 | 2 | 29 | 28 (96.6) |
| Psychiatrist 8 | - | - | - |

* Condition A: delegation of clozapine-monitoring tasks to a trained advanced nurse practitioner.

** Condition B: treatment as usual, clozapine monitoring by a psychiatrist.

*** The psychiatrist instructed the patient to visit a laboratory, for blood tests, but he failed to notice that the patient did not follow this instruction.
