# Supplementary material for: Delegating Clozapine Monitoring to Advanced Nurse Practitioners: An Exploratory, Randomized Study to Assess the Effect on Prescription and Its Safety
Source: Adm Policy Ment Health. 2020 Mar 18;47(4):632–40. doi: 10.1007/s10488-020-01031-4 (PMC7253396; doi:10.1007/s10488-020-01031-4)
Supplement: Supplementary file 3 — Supplementary file3—Online Appendix (DOCX 43 kb) [file 10488_2020_1031_MOESM3_ESM.docx]

**Appendix Adequate dosages, description of the scores on the Clinical Global Impression-Schizophrenia Scale (CGI-SCH), and the decision tree.**

**Adequate dosage, oral medication**

| ***Drug*** | ***Adequate dosage*** |
| --- | --- |
| **Aripiprazole** | **15 mg/d (2)** |
| **Bromperidol** | **4 mg/d** |
| **Flupentixol** | **4 mg/d** |
| **Haloperidol** | **4 mg/d (3)** |
| **Lurasidone** | **40 mg/d (7)** |
| **Olanzapine** | **15 mg/d (2)** |
| **Paliperidone** | **6 mg/d (5)** |
| **Penfluridol** | **40 mg/wk (5)** |
| **Perphenazine** | **16 mg/d (2)** |
| **Pimozide** | **4 mg/d (5)** |
| **Quetiapine** | **400 mg/d (2)** |
| **Risperidone** | **3 mg/d (2)** |
| **Sertindole** | **12-20 md/d (6)** |
| **Sulpiride** | **800 mg/d (5)** |
| **Zuclopenthixol** | **16 mg/d (4)** |
| **Adequate dosage, long lasting injectables** | |
| ***Drug*** | ***Adequate dosage*** |
| **Aripiprazole** | **400mg/4wk (6)** |
| **Bromperidol** | **100 mg/ 4wk** |
| **Fluphenazine** | **50 mg/4 wk (1)** |
| **Flupentixol** | **40 mg/2wk (1)** |
| **Fluspirilene** | **4 mg/wk (1)** |
| **Haloperidol** | **100 mg/4 wk (1)** |
| **Olanzapine** | **210 mg/ 2 wk (6)** |
| **Paliperidone** | **75mg/4 wk (6)** |
| **Risperidone** | **37,5 mg/2 wk (1)** |
| **Zuclopenthixol** | **225 mg/ 3wk (1)** |

1. Moleman P, Birkenhäger T. Praktische Psychofarmacologie 2009. Houten: Bohn Stafleu Van Loghum.
2. Lieberman J et al. New England Journal of Medicine 2005;353(12):1209-1223.
3. Andreasen N et al. Biological psychiatry 2010;67(3):255-262.
4. Van Alphen C et al. Multidisciplinaire richtlijn schizofrenie 2012. Utrecht: De Tijdstroom.
5. http://www.whocc.no/atc_ddd_index/?code=N05A
6. Van Loenen A. Farmacotherapeutisch Kompas 2003. Amstelveen.
7. Loebel A et al. European Psychiatry 2015;30(1):26-31.

N.B. If the dosage was lower or the period shorter, due to untreatable EPS, this counts as adequate treatment .

**Description of the scores on the Clinical Global Impression-Schizophrenia Scale (CGI-SCH).**

1=Normal-not at all ill, symptoms of disorder not present past seven days.

2=Borderline mentally ill-subtle or suspected pathology.

3=Mildly ill-clearly established symptoms with minimal, if any, distress or difficulty in social and occupational function.

4=Moderately ill-overt symptoms causing noticeable, but modest, functional impairment or distress, symptom level may warrant medication.

5=Markedly ill-intrusive symptoms that distinctly impair social/occupational function or cause intrusive levels of distress.

6=Severely ill-disruptive pathology, behavior and function are frequently influenced by symptoms, may require assistance from others.

7=Among the most extremely ill patients-pathology drastically interferes in many life functions; may be hospitalized.

**Decision tree**

| Diagnosis schizophrenia, schizoaffective disorder or psychotic disorder NOS? | No | No further screening of file. |  |  |
| --- | --- | --- | --- | --- |
| Yes |  |  |  |  |
| Already using clozapine? | Yes | No further screening of file. |  |  |
| No |  |  |  |  |
| Previous use of clozapine? | Yes | No further screening of file. |  |  |
| No |  |  |  |  |
| Score of 5 (markedly ill) or higher for positive symptoms on the CGI-SCH? | Yes | Two different antipsychotics used, including a second-generation antipsychotic? | No |  |
| No |  | Yes |  |  |
| Untreatable extrapyramidal side-effects of antipsychotics? | Yes | Both antipsychotics administered in adequate dosage (see appendix) for at least 4 weeks? | No |  |
| No |  | Yes |  |  |
| At least markedly severe tardive dyskinesia or dystonia? | Yes | Minimally 90% of these drugs taken as prescribed (estimated)? | No | No indication for clozapine (yet). |
| No |  | Yes |  |  |
| Suicide attempt or persistent suicidal thoughts (during current use of antipsychotics)? | Yes | Unmet indication for clozapine. |  |  |
| No |  |  |  |  |
| Aggressive behavior (during current use of antipsychotics)? | Yes |  |  |  |
| No |  |  |  |  |
| No indication for clozapine |  |  |  |  |
